# L1CAM and its cell-surface mutants: new mechanisms and effects relevant to the physiology and pathology of neural cells

**DOI:** 10.1111/jnc.12015

**Published:** 2012-12-10

**Authors:** Luigina Tagliavacca, Federico Colombo, Gabriella Racchetti, Jacopo Meldolesi

**Affiliations:** Department of Neuroscience, Vita-Salute San Raffaele University and San Raffaele InstituteMilano, Italy

**Keywords:** high REST PC12 clones, L1 syndrome, L1CAM signaling, neurite outgrowth, NGF, TrkA receptor

## Abstract

The L1 syndrome, a genetic disease that affects 1/30 000 newborn males, is sustained by numerous missense mutations of L1 cell adhesion molecule (L1CAM), an adhesion surface protein active also in transmembrane signaling, essential for the development and function of neurons. To investigate the cell biology of L1CAM, we employed a high RE1-silencing transcription (factor) clone of the pheochromocytoma PC12 line, defective in L1CAM expression and neurite outgrowth. The clone was transfected with wild-type L1CAM and four missense, disease-inducing point mutants encoding proteins distributed to the cell surface. The mutant-expressing cells, defective in adhesion to extracellular matrix proteins and in migration, exhibited unchanged proliferation. The nerve growth factor (NGF)-induced neurite outgrowth was re-established in defective clone cells transfected with the wild-type and the H210Q and I219T L1CAMs mutants, but not in the others. The stimulated outgrowth was confirmed in a second defective PC12 clone over-expressing the NGF receptor TrkA, treated with NGF and/or a recombinant L1CAM chimera. These results revealed a new function of L1CAM, a positive, robust and dose-dependent modulation of the TrkA receptor activated spontaneously or by NGF. The variable effects observed with the different L1CAM mutants suggest that this function contributes to the marked heterogeneity of symptoms and severity observed in the patients affected by the L1 syndrome.

L1 cell adhesion molecule (L1CAM), a member of the immunoglobulin (Ig) adhesion protein superfamily, was first identified in neurons (Salton *et al*. 1983; Ratjen and Schachner 1984) and then in various non-neural cells and tumors, in the brain and outside (reviews: Rawnaq *et al*. 2012; Schafer and Altevogt 2010; Siesser and Maness 2009). The structure of the wild-type (wt) L1CAM includes a large portion exposed to the cell surface, called here the ectodomain, composed of six Ig-like domains and five fibronectin III (FnIII)-like repeats, followed by a single-pass, transmembrane helix and a short cytoplasmic tail. Upon homophilic and heterophilic binding of surface proteins of its own (cis interaction) and adjacent (trans interaction) cells, L1CAM is known to play two major roles, participating in the dynamics of cell adhesion and in the generation of transmembrane signals at tyrosine kinase receptors (Panicker *et al*. 2003; Maness and Schachner 2007; Schmid and Maness 2008; Schafer and Altevogt 2010). During brain development, L1CAM is critical in multiple processes, such as neuronal migration, axonal growth and fasciculation, and synaptogenesis (Kamiguchi 2003; Dityatev *et al*. 2008; Schafer and Altevogt 2010). In the mature brain, it maintains important roles related to the dynamics of neuronal structure and function, including synaptic plasticity (Dityatev *et al*. 2008; Schmid and Maness 2008; Schafer and Altevogt 2010). Finally, in many tumors, including gliomas and glioblastomas, L1CAM does support proliferation and invasiveness, and is thus envisaged as a biomarker of poor prognosis and a target of specific anti-tumor therapy (Suzuki *et al*. 2005; Siesser and Maness 2009; Schafer and Altevogt 2010; Cheng *et al*. 2011).

Studies of the last two decades have lead to the identification of numerous *L1CAM* gene mutations, including many missense point mutations. Many mutations and various truncations were recognized as the cause of a highly heterogeneous neurological syndrome, the L1 syndrome [also known as CRASH (Corpus callosum hypoplasia, mental Retardation, Adducted thumbs, Spastic paraplegia, and Hydrocephalus)], a genetic disease affecting 1 of 30 000 newborn males (Jouet *et al*. 1994; Vits *et al*. 1994; Kenwrick *et al*. 2000; Vos and Hofstra 2010). Depending on the mutation, the disease is of variable severity. Particularly severe are the forms in which the L1CAM protein is knocked down, truncated or trapped within the cell. However, also some mutants that reach the cell surface induce severe syndromes (De Angelis *et al*. 2002; Marx *et al*. 2012; Schafer and Altevogt 2010; Vos and Hofstra 2010; Weller and Gartner 2001). At the moment, therefore, precise criteria correlating the properties of the mutants with the severity of the syndrome are not available.

Over the years, intense investigation of the wtL1CAM and its mutants has been carried out. *L1CAM* knock-out mice have been generated, and some have been investigated also upon knock-in of the gene and/or a few of its mutants. The results have documented interesting defects in the affected mice, corresponding in part to lesions previously reported in L1 syndrome patients (Cheng and Lemmon 2004; Nakamura *et al*. 2010). Additional information was obtained by studies at the cellular level. Murine primary neurons and neuron-like cell lines (N2A, NSC-34), transfected with human *L1CAM* mutants, were found to exhibit altered intracellular trafficking of some encoded forms of the protein (Schafer *et al*. 2010; Itoh *et al*. 2011). In these studies, however, the contribution of the endogenous wtL1CAM, expressed by the neural cells, made the identification of the effects of the transfected mutants difficult. Parallel studies were carried out in transfected non-neural cell lines such as CHO, COS, HEK-293, NIH, and NIH-3T3 fibroblasts (De Angelis *et al*. 2002; Gast *et al*. 2008; Michelson *et al*. 2002; Needham *et al*. 2001; Rünker *et al*. 2003). These lines have the advantage to express no endogenous L1CAM. However, they are profoundly different from neural cells in properties as important as general structure, cytoskeleton architecture and dynamics, membrane traffic, surface molecules, and others.

To extend the investigation of L1CAM and its mutants, we have chosen two clones, PC12-27 and PC12-TrkA, isolated from the neural PC12 cell line that differ from each other in their level of TrkA, the tyrosine kinase receptor of nerve growth factor (NGF) (Leoni *et al*. 1999; Schulte *et al*. 2010). At variance with the other differentiated neural cells including the canonical PC12 cells, the PC12-27 and PC12-TrkA clones are characterized by high levels of the transcription repressor RE1-silencing transcription (factor) (REST) (also known as NRSF) (D'Alessandro *et al*. 2008; Schulte *et al*. 2010), a well-known master factor of neural cell specificity (Ballas and Mandel 2005). As a consequence, their expression of many proteins encoded by REST targets genes, including L1CAM (Bruce *et al*. 2006; Mikulak *et al*. 2012), is repressed, whereas their expression of other gene products and of processes typical of neural cells is normal (Grundschober *et al*. 2002; Schulte *et al*. 2010). The defect of these clones is reversible inasmuch as they reacquire typical neural properties when their REST tone is lowered (D'Alessandro *et al*. 2008; Tomasoni *et al*. 2011). By transient and stable transfection of L1CAM in the two clones, we have investigated the role of wtL1CAM in a variety of neural cell processes, from cell adhesion and migration to proliferation; from TrkA receptor activation to NGF-induced neurite sprouting/outgrowth. Moreover, parallel results obtained with the same clones transfected, however, with one of four L1CAM ectodomain point mutants have revealed differential defects of the above processes. Taken together, our results have provided mechanistic information about the consequences of the mutations. This new information might ultimately be relevant for the understanding of the various forms of L1 syndrome expressed by human patients.

## Materials and methods

### Cell cultures

Canonical PC12 and the two clones, PC12-27 and PC12-TrkA, were previously described (Leoni *et al*. 1999; D'Alessandro *et al*. 2008). Cells were grown in Dulbecco's modified Eagle's medium supplemented with 10% horse serum (Euroclone, Wetherby, UK), 5% fetal clone III serum (Hyclone, Logan, UT, USA), 2 mM l-glutamine, 100 U/mL penicillin and streptomycin (Biowhittaker, Verviers, Belgium). Neurite outgrowth and TrkA autophosphorylation (Y490) were induced by incubation (for 24–48 h and 20 min, respectively) with 5–100 ng/mL of NGF (Alomone, Jerusalem, Israel), and/or with 1–500 ng/mL of the recombinant human, water-soluble chimera including the ectodomain of L1CAM and the Fc domain of human IgGs (L1CAM-Fc) (R&D Systems, Minneapolis, MN, USA) in Dulbecco's modified Eagle's medium supplemented with 1.5% serum. Neurite outgrowth by the ROCK inhibitor Y27632 (Calbiochem, La Jolla, CA, USA), 25 μM, was induced by 1-h incubation of the cells in complete medium. In some experiments, the TrkA inhibitor (Trk-I) Calbiochem 648450, 10 nM; the p75^NTR^ peptide inhibitor (p75^NTR^-I) TAT-Pep5 (Calbiochem), 1 μM; and the neurotrophin antagonist Y1036 (Merck, Nottingham, UK), 30 μM, were used in combination with NGF and/or L1CAM-Fc.

### Stable and transient transfections

The PcDNA3.1 plasmids containing the human cDNA of wt and mutant L1CAMs were a gift of Susan Kenwrick (Cambridge Inst. for Medical Res., Cambridge, UK). Cells were transiently transfected using lipofectamine 2000 (Invitrogen, Carlsbad, CA, USA) and processed 24–48 h later. For stable transfections of PC12-27 cells, the cDNAs of wtL1CAM and mutants were digested out from the PcDNA3 vectors with the restriction enzyme EcoRI and inserted in the EcoRI site of the vector pTracer-CMV/Bsd containing the GFP-blasticidin fusion gene (Invitrogen). Constructs were validated by DNA sequencing. Mock-transfected cells were transfected with the empty vector. Transfected cells were sorted using a FACSorter flow cytometer and grown in the presence of 10 μg/mL of blasticidin S-HCl.

### Western blotting

Cells grown in a low-density monolayer were suspended in lysis buffer containing 1% Triton X-100, 50 mM Tris-HCl (pH 7.5), 250 mM NaCl, 5mM EDTA, phosphatase and protease inhibitors (Sigma-Aldrich, St. Louis, MO, USA). Lysates were cleared by 10-min centrifugation at 1000 *g*, 4°C, separated by 8/12% sodium dodecyl sulfate–polyacrylamide gel electrophoresis and transferred to nitrocellulose filters. The following antibodies (Abs) were used: the goat anti-L1CAM C20 (sc-1508; Santa Cruz Biotec., Santa Cruz, CA, USA) and the rabbit anti-pTrkA (Y490) (Cell Signaling Tech., Danvers, MA, USA). The anti-GAPDH Ab was a mouse monoclonal (Sigma-Aldrich). Blots were developed by chemiluminescence (ECL detection reagent, Amersham Biosci., Little Chalfont, UK). Signals were acquired by a densitometer and quantized using the ImageJ software (NIH, Washington, DC, USA).

### Immunofluorescence and surface biotinylation

Cells were grown at low density (no more than 40% confluency) on poly-l-lysine-coated coverslips, fixed with 4% formaldehyde/phosphate-buffered saline (PBS), and incubated with the goat anti-L1CAM Ab N14 (sc-31032, Santa Cruz Biotec) addressed to the ectodomain of the adhesion protein. The FITC-conjugated anti-goat IgG secondary Ab (Sigma-Aldrich) was then applied without permeabilization. Alternatively, the cells were first permeabilized with 0.4% Triton X-100 in PBS with 1% bovine serum albumin and then treated with either a polyclonal [the goat anti-L1CAM-C20 (Santa Cruz Biotec); the rabbit anti-calnexin (SPA-860, Stressgen Bioreagents, Victoria, BC, Canada)]; or a mouse monoclonal [anti-human L1CAM (BD Biosci. Pharmingen, San Diego, CA, USA)]; anti-β-tubulin (Sigma-Aldrich); anti-giantin (US Biological, Boston, MA, USA) Abs. Coverslips were rinsed and incubated with the appropriate secondary Abs, TRITC- or FITC-conjugated, and nuclei were stained with 4′-6-diamidino-2-phenylindole (DAPI) (Sigma-Aldrich). Samples were studied using Biorad MRC 1024 (Microscopy at UW-Madison, Madison, Wis, USA) or using wide-field Olympus IX70 microscope (N.Y. Microscope, Hicksville, N.Y., USA) with DeltaVision RT Deconvolution. Images of at least 150 neurites/group were outlined manually and their lengths measured using the ImageJ software. Two types of information were obtained from the measurements: percent of cells exhibiting one or more neurites > 10 μm in length, and average length of the neurites expressed by single cells.

For biotinylation of plasma membrane proteins (Borgonovo *et al*. 2002), petri dishes of PC12-27 cells, stably transfected with wtL1CAM or one of the four mutants, were washed three times with ice-cold PBS before 30-min incubation at 10°C with 0.1 mg/mL sulpho-NHS-LC-biotin (Pierce, Rockford, IL, USA). Cells were then washed twice with cold PBS and resuspended in lysis buffer supplemented with proteases inhibitors. Most nuclei were removed by low-speed centrifugation at 4°C (1000 *g*, 5 min). The supernatants were mixed with an equal volume of streptavidin–agarose beads (Pierce) and gently stirred for 1 h at 4°C. The biotin–streptavidin complexes, separated from unbound fractions by centrifugation, were then analyzed by western blotting. The viability of the analyzed cells was documented by the lack, in the biotin–streptavidin complex preparations, of the cytosolic enzyme glyceraldehyde-3-phosphate dehydrogenase assayed in the cell lysate. Data of biotinylated L1CAM are expressed as percentage of total L1CAM.

### Separation of detergent-resistant and detergent-solubilized plasma membrane domains

Canonical PC12 and variously transfected PC12-27 cells, suspended in 0.32 M sucrose/5 mM Hepes, pH 7.4 with protease inhibitors, were gently homogenized in a cell cracker and then centrifuged at 1000 *g* for 5 min. Upon resuspension and recentrifugation, the low-speed pellets, enriched in plasma membrane fragments, were suspended in ice-cold sucrose/Hepes solution supplemented with 0.5% Triton X-100. After 30 min on ice, the preparations were diluted to a final sucrose concentration of 42% and applied at the bottom of a small centrifugation tube that was covered with two cushions (of 38 and 5% sucrose) in a discontinuous floatation gradient. Upon centrifugation at 250 000 *g* for 18 h, the detergent-resistant (DR) membranes were recovered in the band floating over the 38% sucrose cushion; the detergent-solubilized (DS) membranes in the 42% sucrose-loading cushion. For further details, see Cocucci *et al*. (2007). Aliquots (100 μL) of the two gradient fractions were analyzed by parallel dot-blot assays with the following Abs: anti-L1CAM (sc-1508, Santa Cruz Biotec); anti-transferrin receptor (TfR), a marker for DS membrane (US Biological, MA, USA); and anti-CD90/Thy1.1, a marker for DR membranes (Serotec, Kidlington, UK). Blot images were acquired using Image J. The data were expressed as percentage of the recovered L1CAM, TfR, and CD90/Thy1.1, present in the DR membrane fraction.

### Adhesion assays

Adhesion assays were performed in 96-well plates coated with collagen IV or Fn (BD Bioscience), 20 μg/mL, or a combination of the two, dissolved in PBS. The plates were incubated at 4°C overnight, then rinsed, blocked with 0.2% bovine serum albumin for 2 h at 37°C, and washed three times in PBS. Thirty thousand canonical PC12 or PC12-27 cells, transfected or not with the various forms of L1CAM, were applied to each well in quadruplicate and incubated for 1 h at 37°C. After washing, the cells attached to the plates were fixed with 4% formaldehyde and stained for 25 min at 20°C with a solution containing 0.5% crystal violet, 2% ethanol, and 40% methanol in PBS. Wells were washed with water and cells were solubilized with 100 μL of 1% SDS. Color absorbance was measured at 540 nm with a microplate reader. The percentage bound cells was calculated from the ratio between the optical density of the adherent cells and that of the mock cells processed in parallel, taken as 100%.

### Scratch assay of migration

PC12-27 cells, stably transfected with wtL1CAM or with single mutants, were seeded on uncoated six-well plates and cultured until confluence. A straight scratch of the monolayer was made by hand using a P200 tip, and incubation of the cells was pursued for 3 days at 37°C upon switching to a starvation medium containing 1.5% serum. Images of several sites of the scratch, chosen at random, were taken every day using a Zeiss microinjection microscope (Carl Zeiss AG, Oberkochen, Germany) with a 25X phase contrast objective. The migration of the cells in each preparation was deduced from the changes of the scratched areas at the various investigated sites, measured using the ImageJ software. Specifically, the difference between the various areas at the times of analysis with respect to the same areas at time 0 was divided by the latter and taken as the migration value.

### Cell proliferation

Proliferation of the variously transfected PC12-27 cells was investigated by two complementary assays. The 3-(4,5-dimethylthiazol-2-yl)-2,5-diphenyltetrazolium bromide (MTT) assay, to reveal the changes in the number of living cells in the various transfected PC12-27 cell populations, was performed in five parallel sets of 96-well plates. Thousand cells in 200 μL of serum-supplemented medium were seeded in each well. Twenty-four hours after seeding and then at 24-h intervals, single sets of wells were treated with 20 μL of 5 mg/mL MTT (Sigma–Aldrich) for 2 h, after which the medium was replaced with 100 μL of dimethylsulfoxide, and the absorbance was recorded at 570 nm using a 96-well plate ELISA reader.

The Ki67 assay was carried out to reveal cells in the G1-M phases in the various transfected PC12-27 populations. Two thousand cells in 100 μL of serum-supplemented medium were seeded in triplicate in 96-well plates. The next day, and then 24 and 48 h later, sets of cells were fixed with 4% formaldehyde, permeabilized with 0.4% Triton X-100 in PBS and stained with a rabbit polyclonal anti-Ki67 Ab (Thermo Fischer Scientific, Milan, Italy), followed by a goat anti-rabbit, TRITC-conjugated Ab, revealing the nuclei of competent cells. All nuclei were then stained with DAPI. Images were taken using the INCell Analyzer 1000 microscope (GE Healthcare, UK). Competence was established from the ratio between Ki67-positive and DAPI-stained nuclei, counted in parallel using the INCell Investigator Software (GE Healthcare, Milan, Italy).

### Statistical analyses

The significance of the data was assessed using the two-tailed unpaired *t*-test and the anova test. Data shown are averages ± standard deviations (SD). The number of experiments is specified in the figures or legends.

## Results

### Expression of L1CAM in PC12-27 cells transfected with the wt or mutant cDNAs

**Fig. 1:**
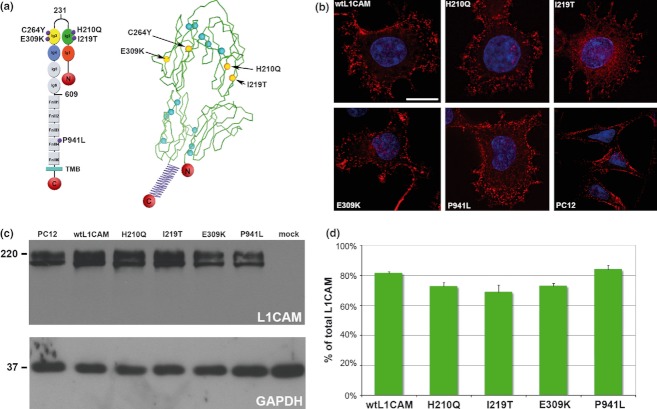
Structure and expression of L1 cell adhesion molecule (L1CAM) in stably transfected PC12-27 cells. (a) The general and the tridimensional structure of wtL1CAM (Arevalo *et al*. 2008; Liu *et al*. 2011) showing the localization of four investigated mutations, two in the second and two in the third immunoglobulin (Ig) domain. The fifth mutation, in the third fibronectin III (FnIII)-like repeat, is shown in the left but not in the right drawing. (b) L1CAM immunocytochemistry (red) showing the whole-cell distribution of the stably transfected wt and mutant L1CAM in PC12-27 cells, and of the endogenous protein expressed by canonical PC12 cells. The nuclei were stained blue with 4′-6-diamidino-2-phenylindole (DAPI). The images shown are representative of at least 25 images from three experiments. The bar on the upper left, valid for all (b) panels, is of 10 μm. (c) Western blot of L1CAM (representative of eight experiments). In the mock-transfected cells (right) the level of the protein is below detection; in those transfected with the wtL1CAM and mutants the levels of the protein (that appears in all cases as two bands of 200–220 KDa apparent molecular weight) are either comparable (E309K and P491L) or moderately higher (wtL1CAM, H210Q, and I219T) than those of the endogenous protein in the canonical PC12 cells (left). With the development time used with this blot, no bands corresponding to low molecular weight fragments of L1CAM are seen. (d) Percentage of the total wt and mutant L1CAM protein, stably transfected in PC12-27 cells, which is exposed to the cell surface, as shown by surface biotinylation (± SD of three experiments).

The model of Fig. 1a illustrates the general organization of the wtL1CAM protein (Arevalo *et al*. 2008; Liu *et al*. 2011) and the distribution of four of the five point mutations investigated here, all located in the ectodomain of the protein. Two of these mutations, H210Q and I219T, localized in the second Ig-like domain, emerge at the surface of the N-terminal loop, whereas E309K and C264Y, in the third Ig-like domain, are located more deeply in the folded protein. The fifth mutation, P941L, resides in the forth FnIII-like repeat, in the proximity of the transmembrane domain (not shown).

In the initial experiments, the high REST cells of the PC12-27 clone, which express very low levels of endogenous L1CAM (Mikulak *et al*. 2012), were transfected transiently with either the wtL1CAM or its ectodomain point mutant cDNAs. Other cells were mock transfected with the empty vector. Based on previous studies on neurons (Michelson *et al*. 2002; Cheng and Lemmon 2004), transfection of the various L1CAM forms was expected to induce some differential neurite outgrowth responses. The Figure S1a reports the phenotype of typical, transiently transfected cells and compares their surface (left panels) and total (right panels) L1CAM immunolabeling, as revealed by anti-L1CAM Abs administered to the cells before or after detergent permeabilization, respectively. Most transfected cells appeared stellate for the presence of few short and thick processes. Typical thin neurites were in contrast, almost completely absent. In the non-permeabilized cells (Figure S1a, left panels), the surface distribution of wtL1CAM and of the four mutants H210Q, I219T, E309K, and P941L appeared even, covering both the cell body and the thick processes. In contrast, in the cells expressing the C264Y mutant, almost no L1CAM was detected exposed at the cell surface. These differences among the transfected clones were no longer visible in the transfected cells that had been detergent permeabilized before immunolabeling to reveal their total L1CAM level (Figure S1a, right panels). To identify the intracellular structure where the transfected C264Y mutant was retained, we carried out dual immunolabeling of the permeabilized cells employing, in addition to L1CAM, either the endoplasmic reticulum (ER) marker calnexin or the Golgi marker giantin. Figure S1b shows that a colocalization of L1CAM is clear with calnexin (upper row), whereas with giantin it is marginal. Also in the case of the other mutants, a fraction of L1CAM, however much smaller, was found labeling the ER (not shown). The L1CAM mutant retained intracellularly might be destined to degradation by the ER quality-control system.

A detailed study of the surface-exposed wt and mutated L1CAMs was carried out in stably transfected PC12-27 cells sorted by FACS from non-transfected cells. In view of the interest of the work in the surface L1CAM forms, in these experiments and in the rest of the article, the transfected C264Y mutant was no longer investigated. The immunocytochemistry shown in Fig. 1b shows that the cells transfected with the wtL1CAM or with one of the four other mutants maintain the flat phenotype typical of PC12-27 cells, different from the smaller and convex phenotype of the canonical PC12 (Tomasoni *et al*. 2011). The distribution of the various L1CAM forms was moderately different, with higher concentration at the periphery in the wt, H210Q- and E309K-transfected cells. The western blot of Fig. 1c shows that, in PC12-27 cells mock transfected with the empty plasmid, L1CAM was almost undetectable as in the non-transfected PC12-27 previously investigated by Mikulak *et al*. (2012). In PC12-27 cells expressing the transfected L1CAMs, in contrast, the levels of the adhesion protein varied from values close to the canonical PC12 cells (the E309K and P491L mutants) to values 25–30% higher (the wtL1CAM and two, H210Q and I219T, mutants). In the lysates from all analyzed cells, two L1CAM bands could be detected at ∼ 200–220 kDa (Fig. 1c). The two bands could be because of L1CAM molecules distinct by their differential glycosylation state (see Ratjen and Schachner 1984; De Angelis *et al*. 2002; Schafer *et al*. 2010). In the conditions of the experiments, proteolytic fragments of L1CAM with smaller molecular weight could not be detected in the rest of the gel, except when the exposure of the blots was prolonged. Also in this case, the contribution of the fragments appeared marginal.

The surface distribution of L1CAM stably transfected in PC12-27 cells was further investigated by biotinylation to obtain quantitative data. Figure 1d shows that the surface fraction accounted for slightly over 80% of the total L1CAM protein expressed by the wtL1CAM-transfected cells. The value found in the cells transfected with the P941L mutant (82.5%) was not significantly different from the wtL1CAM. Those of H210Q and E309K (72%) and of I219T (67%) were lower, but moderately (Fig. 1d). Taking into account the differences in the total levels of expression of the various forms (Fig. 1c), we conclude that the surface levels of the protein in the PC12-27 cells transfected with one of the four mutants varied from approximately 70% (E309K) to 86% (H210Q) with respect to the wtL1CAM-transfected cells, that is, they were lower but still considerable.

### Distribution of the various L1CAM forms, wt and mutants, in raft and non-raft microdomains of the plasma membrane

The plasma membrane is known to be heterogeneous in its molecular and structural organization. In particular, various surface proteins, including L1CAM (Tang *et al*. 2011), are known to concentrate in microdomains rich in cholesterol, the so-called rafts, with possible consequences in terms of function (Lingwood and Simons 2010). To investigate the distribution of wtL1CAM and its mutants in the raft and non-raft microdomains, we carried out experiments of controlled plasma membrane treatment with a non-ionic detergent, and analyzed the two fractions obtained, the detergent-soluble (DS) and detergent-resistant (DR) fractions. Moreover, to validate the results with L1CAM, we compared them with those of two endogenous markers investigated in parallel, TfR and CD90/Thy1.1, known to concentrate in the DS and DR plasma membrane domains, respectively. Figure S2 illustrates the recovery of the transfected L1CAM forms and of the two markers in the DR fraction, isolated from a low-speed pellet enriched in plasma membranes, first exposed to Triton X-100 (0.5%, 30 min, 10°C) and then recovered by high-speed, step-gradient centrifugation in a band floated at the interface between the 38% and the 10% sucrose cushions (Cocucci *et al*. 2007). As can be seen, the CD90/Thy1.1 and the TfR markers exhibited in all analyzed cells the expected distribution, with large and only marginal (background) accumulation in the floated DR band, respectively. The DR accumulation of the various L1CAM forms transfected in PC12-27 cells was moderate and variable. The values of the I219T- and P941L-transfected cells were distinctly higher, those of H210Q- and E309K–transfected cells were lower than those of wtL1CAM-transfected cells. However, in all cases the L1CAM values were significantly higher than the background levels of TfR. These results confirm some accumulation of L1CAM in the rafts and suggest that the mutations modify this process, however, variably. Compared with the canonical PC12, the DR accumulation of the wtL1CAM from the transfected PC12-27 cells was < 40% (Figure S2), suggesting that the raft affinity of the adhesion protein depends only in part on its own properties and for the rest on its interaction with other membrane component(s) present in the canonical PC12, which are reduced or lacking in the PC12-27 cells. This issue, which could have some functional importance (Chang *et al*. 2006; Lingwood and Simons 2010; Tang *et al*. 2011), remains to be further investigated.

### Functional assays: cell adhesion, migration, and proliferation

**Fig. 2:**
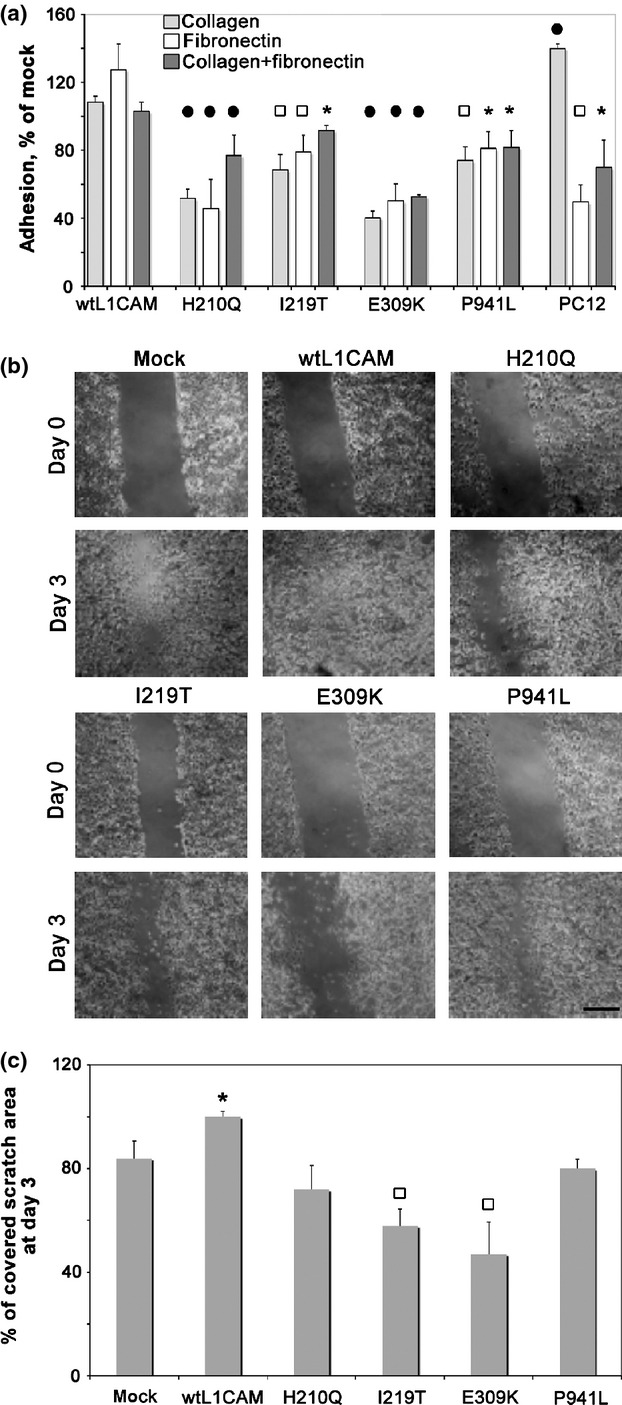
Adhesion to cell-matrix proteins and migration of transfected PC12-27 cells. (a) In PC12-27 cells stably transfected with wild-type L1 cell adhesion molecule (wtL1CAM), adhesion is slightly higher than in mock-transfected PC12-27 cells. With respect to canonical PC12 cells (right), adhesion of wtL1CAM transfected cells to Fn and Fn+collagen IV is much higher than that to collagen IV. In contrast, PC12-27 cells transfected with the various L1CAM mutants (especially E309K) exhibit significantly lower adhesion to all three matrix components (averages ± SD of 12 experiments). The marks ★, □, and • on top of the histograms indicate significance (*p* < 0.05, < 0.01, and < 0.001, respectively) relative to mock-transfected cells. (b) Scratch test of migration performed on confluent monolayers of cells seeded directly on plastic. The bar on the bottom right = 100 μm. (c) Percent quantization of the scratch assay results (averages ± SD of 12 experiments). Wound areas at day 3, measured at 10 sites of the scratch chosen at random were subtracted from the areas measured at the same sites at time 0, taken as 100%. Values shown in (c) are averages of the cell migration data at day 3. Statistical marks in (c) as in (a).

In view of the role of L1CAM in cell adhesion, expression of its wt and mutated forms was expected to affect the results of specific assays carried out on surfaces coated with extracellular matrix proteins. To investigate this possibility, stably transfected PC12-27 cells were seeded on plates coated with either collagen IV, Fn, or the two together, and their adhesion was measured by a colorimetric assay. As shown in Fig. 2a, the PC12-27 cells transfected with wtL1CAM exhibited levels of adhesion slightly higher than those of the mock-transfected cells. In contrast, the adhesion of the cells transfected with the mutated L1CAMs was lower than that of mock-transfected cells, moderately in the case of the I219T and P941L mutants, more markedly in the case of the H210Q and, even more, of the E309K mutants. Taken together, the results confirm that wtL1CAM participates in cell adhesion, as expected. This property, however, is lost by mutants, all of which do not increase but repress adhesion significantly. With respect to canonical PC12 cells, adhesion of wtL1CAM-transfected PC12-27 cells was different, higher on Fn and lower on collagen IV (Fig. 2a). As in the case of the raft accumulation (Figure S2), these differences might be because of surface molecules other than L1CAM, present in the canonical PC12 and lacking in PC12-27 cells. Therefore, they will not be investigated here any further.

The differences in adhesion among stably transfected PC12-27 cells were complemented by the results of a scratch assay of cell migration (Fig. 2b and c). The conditions of these experiments were different from those of adhesion because migration occurred on uncoated plastic surfaces, yet the results were consistent. PC12-27 cells transfected with wtL1CAM migrated significantly faster than cells transfected with mutant L1CAM. Thus, the scratched gap was closed quicker by the PC12-27 cells transfected with wtL1CAM than by PC12-27 cells transfected with the L1CAM mutants. The delay was relatively small for the cells expressing the H210Q and P941L mutants and highest for those expressing the E309K mutant, that is, the cells exhibiting also the lowest adhesion (Fig. 2a). Specifically, when the scratch of the wtL1CAM monolayer was no longer visible, the scratch of the E309K monolayer still exhibited ∼50% of its initial area (Fig. 2b and c).

A third cell function, proliferation, was investigated in stably transfected PC12-27 cells by applying two complementary assays. The MTT assay, which reveals the number of living cells, can be interpreted in terms of proliferation only if cell senescence and death are marginal. The Ki67 assay, on the other hand, labels the nuclei of cells undergoing the G1–M phases. Figure S3a shows that the labeling with MTT rose with time in parallel in the PC12-27 cells mock transfected and transfected with the various L1CAM forms investigated. In the Ki67 assay, on the other hand, the % labeled nuclei in mock and L1CAM-transfected cell populations were not significantly different, accounting on the average for over 95% of the total DAPI-positive nuclei (Figure S3b, c). These results exclude the MTT data to be affected by senescence and death of the cells. We conclude that at variance with non-neural cells (Suzuki *et al*. 2005; Siesser and Maness 2009; Schafer and Altevogt 2010), L1CAM plays no major regulatory role in the proliferation of the neural PC12-27 cells.

### Neurite outgrowth

A typical property of canonical PC12 cells, induced by day-long treatment with NGF, is neurite outgrowth accompanied by the arrest of proliferation and the acquisition of neuron-type markers (Greene and Tischler 1976). Previous studies had established this response to be greatly attenuated in PC12-27 cells unless transfected with the high-affinity NGF receptor, TrkA (Leoni *et al*. 1999; Schulte *et al*. 2010). In contrast, when the cells were treated not with NGF, but with blockers of ROCK, an effector kinase of the small GTPase RhoA, neurite outgrowth occurred in PC12-27 cells more rapidly than in canonical PC12 (Racchetti *et al*. 2010). The task of our experiments was to establish whether, in loosely seeded PC12-27 cells, expression of L1CAM or its mutants affect neurite outgrowth induced by NGF and ROCK blockers.

**Fig. 3:**
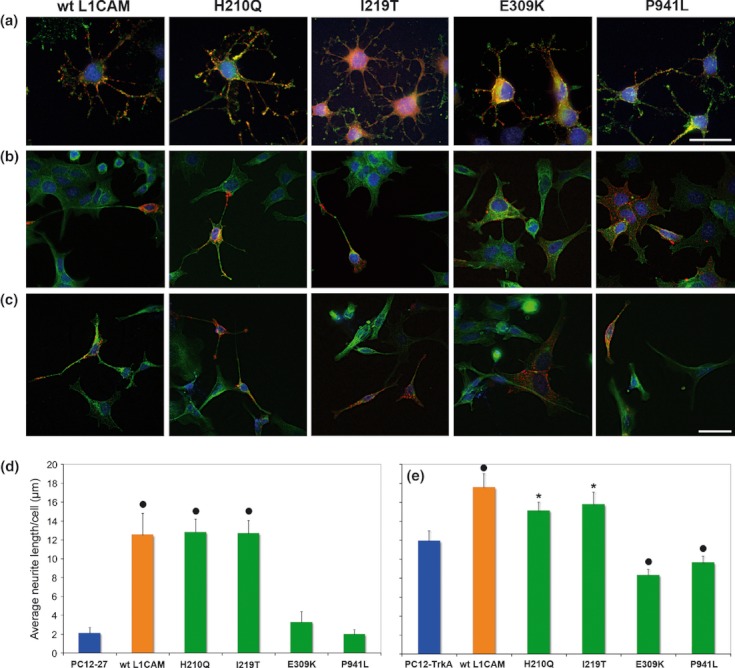
Neurite outgrowth induced by treatment with Y27632 (a) and nerve growth factor (NGF) (b–e) in PC12-27 and PC12-TrkA cells non-transfected and transfected with various forms of L1 cell adhesion molecule (L1CAM). A: Immunofluorescence (red = L1CAM; green = β-tubulin; blue = DAPI) of PC12-27 cells stably transfected with wt and mutated L1CAMs, treated for 60 min with Y27632 (25 μM). The images shown are representative of at least 18 images from three experiments. The bar on the right, valid for all A panels, is of 20 μm. (b) Immunofluorescence as in (a), however, of PC12-27 cells transiently transfected with wtL1CAM and mutants, treated with NGF (100 ng/mL) for 48 h. Quantization of the results in (d) (histograms are averages of > 150 cells from three experiments ± SD). (c) Immunofluorescence as in (a), however, of PC12-TrkA cells transiently transfected with wtL1CAM and mutants, treated with NGF as in (b). Quantization of the results in (e) (histograms are averages of > 150 cells from three experiments ± SD). The bar in panel (c) right, valid for all panels of (b and c), is of 20 μm. Significance of quantitative changes observed with respect to non-transfected PC12-27 and PC12-TrkA [left columns in (d) and (e)] is indicated as in Fig. 2a.

Figure 3a shows that 1-h treatment with the ROCK blocker Y27632 (25 μM) induced, in PC12-27 cells stably transfected with wtL1CAM, extensive outgrowth of thin neurites analogous to those previously observed in the non-transfected PC12-27 cells (Racchetti *et al*. 2010). Among the cells transfected with L1CAM mutants, those transfected with H210Q or I219T exhibited numerous and long neurites similar to those of the cells transfected with the wtL1CAM; those transfected with E309K or P941L exhibited less numerous and shorter neurites (Fig. 3a). Differences among the variously transfected PC12-27 cells emerged also in the surface distribution of L1CAM. In the cells transfected with the wt or with the two mutants, H210Q and P941L, the adhesion protein was distributed primarily over the neurite arborizations, whereas in those transfected with the I219T or the E309K mutants it appeared also over the cell body (Fig. 3a). We conclude that neither wtL1CAM, nor its mutants stimulate or block the rapid outgrowth process triggered by ROCK inhibition first reported in non-transfected PC12-27 cells (Racchetti *et al*. 2010). However, the mutants can induce changes in the number/length of the neurites and exhibit differences in their cell surface distribution.

The subsequent series of experiments was carried out by using in parallel the two high REST clones, PC12-27 and PC12-TrkA, transiently transfected with the various forms of L1CAM. The large difference in TrkA expression of the two clones has been already reported (Leoni *et al*. 1999; Schulte *et al*. 2010). Without NGF, PC12-27 cells transfected with the various L1CAMs did exhibit no thin neurites, but only short and thick processes emerging from the cell body, as already shown in Figure S1a. Likewise, in the L1CAM-negative, non-transfected or mock-transfected PC12-27 cells, 48-h treatment with NGF induced no significant change of shape (not shown; see Leoni *et al*. 1999). In contrast, in the PC12-27 cells transfected with wtL1CAM or its H210Q and I219T mutants, the same treatment with NGF induced outgrowth of few, but thin and long (mean > 12 μm) neurites, ending up with typical growth cones (Fig. 3b and d). This picture was completely different in the PC12-27 cells transfected with the E309K and P941L mutants which, upon NGF treatment, exhibited several thick and only rare thin expansions (Fig. 3b and d), thus resembling the cells transfected with wtL1CAM not treated with NGF (Figure S1a).

Experiments with NGF were carried out also using PC12-TrkA cells. Because of their high density of TrkA receptors, these cells exhibit a partially differentiated phenotype, with thin neurites grown already without NGF treatment. Upon NGF treatment, this phenotype is further developed (Leoni *et al*. 1999; Schulte *et al*. 2010). In PC12-TrkA cells transiently transfected with the various L1CAM forms, but not treated with NGF, the phenotype was not significantly different from that of the non-transfected cells (Fig. 3e and not shown). In contrast, transfection with the various L1CAM forms was found to affect differentially the NGF-induced responses. Figure 3c and e show that, in PC12-TrkA cells transfected with wtL1CAM or its mutants of the second Ig-like domain, H210Q and I219T, the neurites induced by NGF (100 ng/mL) were significantly longer than those of the non-transfected (Fig. 3e) and mock-transfected (not shown) PC12-TrkA cells. In contrast, in the PC12-TrkA cells transfected with the E309K and P941L mutants and then treated with NGF, neurites were few and even shorter than those of the non-transfected PC12-TrkA cells (Fig. 3c and e).

The results obtained by NGF treatment of the two, PC12-27 and PC12-TrkA clones, characterized by different levels of TrkA, although expectedly different, appeared nevertheless consistent with each other (compare Fig. 3b and d to c and e). Reinforcement of the responses induced by NGF was in fact evident in the clones transfected with wtL1CAM or with its two second Ig-like domain mutants. In the clones transfected with the other two mutants, in contrast, the NGF-induced neurite outgrowth responses were unchanged or decreased.

### Effects of the recombinant L1CAM-Fc protein

**Fig. 4:**
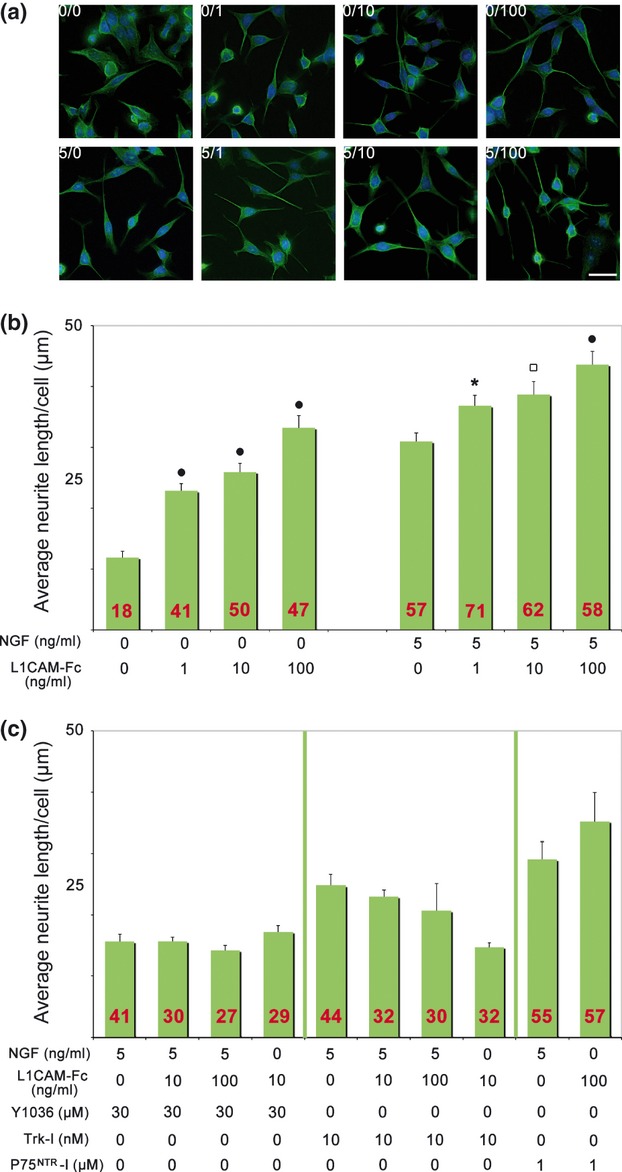
Dose-dependent neurite outgrowth induced by nerve growth factor (NGF) and/or recombinant, water-soluble chimera including the ectodomain of L1CAM and the Fc domain of human IgGs (L1CAM-Fc) in PC12-TrkA cells. Effects of inhibitory drugs. (a) The immunofluorescence of β-tubulin [green; nuclei labeled blue by 4'-6-Diamidino-2-phenylindole (DAPI)] illustrates the phenotype of PC12-TrkA cells treated for 24 h with increasing concentrations of L1CAM-Fc alone (upper row) or together with a small concentration (5 ng/mL) of NGF (lower row), as specified by the white numbers that appear over the panels. The bar on the bottom right, valid for all (a) panels, is of 20 μm. (b) Left and right, quantization of the results taken from the data illustrated in (a), upper and lower rows: average neurite lengths ± SD (> 150 cells per sample from three experiments/histogram; significance calculated with respect to the data without L1CAM-Fc- marked as in Fig. 2a); and % cells exhibiting thin neurites, > 10 μm long, specified by the numbers written in red over the histograms. (c) Effects on neurite outgrowth and on the % cells with thin neurites [marked as in (b)] induced in PC12-TrkA cells by the NGF-binding drug Y1036 (left), the TrkA inhibitor Calbiochem 648450 (Trk-I, middle), and the p75^NTR^ peptide inhibitor TAT-Pep5 (p75^NTR^-I, right), administered at the indicated concentrations 1 h before NGF and/or L1CAM-Fc application, and then maintained together with the latter for 24 h. To evaluate the effects of the drugs, compare the individual data in (c) with the corresponding data with and without L1CAM-Fc shown in (b).

The results of Fig. 3 suggest L1CAM to play an important role in the neurite outgrowth responses induced by NGF, a role that is maintained by some of its mutants, but not by others. However, the results say nothing about where and how this role is played. To establish whether this occurs at the surface of the cells, experiments were carried out by the use of L1CAM-Fc (Roonprapunt *et al*. 2003), a human chimeric, water-soluble recombinant protein including the L1CAM ectodomain fused to the Fc domain of human IgGs. In PC12-TrkA cells, rich in TrkA but lacking L1CAM, long-term treatment with the recombinant protein induced highly significant, dose-dependent neurite outgrowth responses accompanied by an increase in the percentage of cells exhibiting neurites longer than 10 μm (indicated by the numbers drawn in red over the histograms), appreciable already at 1 ng/mL and reaching considerable levels at 100 ng/mL (Fig. 4a, upper line; Fig. 4b, left). When L1CAM-Fc was combined with a small concentration of NGF (5 ng/mL), the dose-dependent neurite outgrowth induced by L1CAM-Fc in PC12-TrkA cells over that of the cells receiving NGF alone was smaller, but still evident and significant (Fig. 4a, lower line; Fig. 4b, right). When in contrast L1CAM-Fc was applied alone to either canonical PC12 or PC12-27 cells, it failed to induce any significant effect (not shown).

The L1CAM-Fc-induced responses of Fig. 4a–b could depend on the constitutive activation of the TrkA receptor typical of PC12-TrkA cells. To investigate this possibility, neurite outgrowth was investigated in PC12-TrkA cells pre-treated with drugs known to affect the NGF signaling: Y1036, which binds NGF, preventing its interaction with the TrkA receptor (Eibl *et al*. 2010); Trk-I, the Calbiochem 648450 inhibitor of TrkA (Lazaridis *et al*. 2011); or p75^NTR-^I, the TAT-Pep5 inhibitor of p75^NTR^ (Yamashita and Tohyama 2003). Fig. 4c left subpanel illustrates the responses in the cells pre-treated with Y1036. The increase of both neurite length and number of responsive cells, observed upon treatment with NGF (5 ng/mL), L1CAM-Fc (10 ng/mL), or the two together, as shown in Fig. 4b left, was almost completely eliminated. Also, Trk-I pre-treatment affected considerably the responses to NGF and, even more, to L1CAM-Fc in terms of both neurite length and percent neurite-exhibiting cells (Fig. 4c, middle). In contrast, the p75^NTR^ TAT-Pep5 inhibitor (p75^NTR^-I, 1 μM) did not change neurite outgrowth, no matter whether induced by NGF or L1CAM-Fc, and increased the percent of neurite-exhibiting cells induced by L1CAM-Fc (Fig. 4c right).

**Fig. 5:**
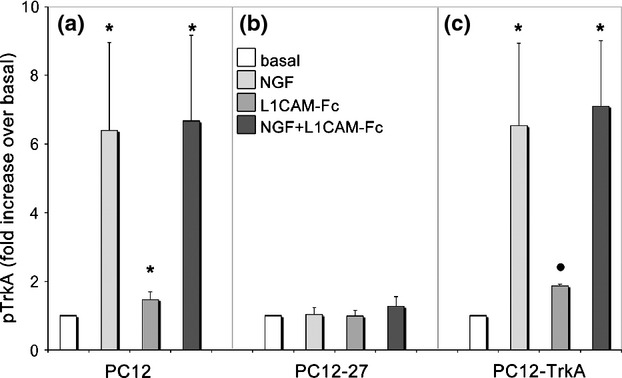
Phosphorylation of the TrkA receptor at the Y490 site induced by nerve growth factor (NGF) and recombinant, water-soluble chimera including the ectodomain of L1CAM and the Fc domain of human IgGs (L1CAM-Fc) in canonical PC12, PC12-27, and PC12-TrkA cells. Parallel aliquots of canonical PC12 cells (a), PC12-27 (b), and PC12-TrkA cells (c) were exposed for 20 min to NGF (50 ng/mL), L1CAM-Fc (500 ng/mL), or the two together. Y490 phosphorylation of TrkA was analyzed in western blots stained with the specific anti-pTrkA Ab. The values shown were calculated with respect to the constitutive phosphorylation level of TrkA (basal), which in PC12-27 cells was 10% and in PC12-TrkA was 300% with respect to the canonical PC12. In each panel, the significance of the differences (with respect to the basal phosphorylation levels) is marked as in Fig. 2a.

In a further investigation, we checked whether L1CAM-Fc had any effect on an early step of NGF signaling, TrkA tyrosine phosphorylation at the Y490 site. Canonical PC12, PC12-27, and PC12-TrkA cells were exposed in parallel for 20 min to either NGF (50 ng/mL), L1CAM-Fc (500 ng/mL), or the two together. Figure 5a shows that in the canonical PC12, where constitutive phosphorylation of the TrkA Y490 site is relatively low and where NGF induced a six-fold increase, L1CAM-Fc administered alone induced a much smaller but significant increase, and failed to induce a significant reinforcement of the response induced by NGF when applied together. In PC12-27, where constitutive phosphorylation is only 10% with respect to canonical PC12 (not shown), neither NGF nor L1CAM-Fc induced any detectable increase of Y490 phosphorylation, while the combination induced only a very small, non-significant increase (Fig. 5b). Finally, in PC12-TrkA cells, where the basal Y490 phosphorylation is large (three-fold the canonical PC12, not shown), documenting the high constitutive autophosphorylation of the receptor, the changes induced by NGF and L1CAM-Fc were similar to those of canonical PC12 when expressed relative to the basal values (Fig. 5c). In absolute terms, however, they were three-fold larger (not shown), as expected in view of the large density of TrkA receptors existing in these cells. Taken together, the data with L1CAM-Fc administered with and without NGF to canonical PC12 and, especially, to PC12-TrkA cells, illustrated in Fig. 4 and 5, strongly suggest the role of L1CAM in neurite outgrowth to consist in a positive modulation of TrkA signaling, taking place upon receptor activation, no matter whether constitutive or induced by NGF.

## Discussion

In this study, the investigation of L1CAM and of four of its missense point mutants has been carried out in two PC12 cell clones, PC12-27 and PC12-TrkA (Leoni *et al*. 1999; Borgonovo *et al*. 2002) that express spontaneously high levels of the transcription repressor REST. The high REST PC12 cells of our model combine three properties advantageous for our study, that is, their features common to neurons, the cells mainly affected by the L1CAM mutations; their very low levels of endogenous L1CAM, critical to distinguish the effects of the transfected wt protein and mutants; and their marked differences in terms of TrkA expression and responsiveness to NGF that has favored the study of L1CAM as a signaling coreceptor.

The preliminary characterization of the PC12-27 clones stably transfected with wtL1CAM and with its mutants revealed a few additional interesting properties of our model. The levels of the various forms of transfected L1CAM were not the same. Of the transfected PC12-27 clones, three, wtL1CAM, H210Q and I219T, exhibited very close levels of L1CAM. The levels of the other two, E309K and P941L, were lower of 25–30% and close to those of the canonical PC12. These differences, which are moderate, exclude our results to be affected by artifacts because of large over-expression or under-expression of the protein in the transfected L1CAM clones. Also, the differences in surface expression of the various transfected forms, revealed by biotinylation, were moderate. Finally, the fraction of the total surface L1CAM recovered in the DR fraction (the rafts) of the plasma membrane, a property of possible functional relevance (Lingwood and Simons 2010; Tang *et al*. 2011), was in all cases small (∼20%), with two mutants higher and two lower than the wt form. Taken together, these results suggest that if considered with caution, the various forms of transfected high REST PC12 cells could be a new and appropriate model for the study of L1CAM.

By the use of our high REST PC12 cell model, we have obtained two series of original results. The first series concerned the functioning of PC12 cells. In the case of proliferation, the values obtained with the cells transfected with the various L1CAM forms were close to those of the mock-transfected PC12-27. This result was unexpected because in numerous non-neural tumors including gliomas, glioblastomas, and neuroendocrine tumors, L1CAM had been found to stimulate proliferation and aggressiveness (Suzuki *et al*. 2005; Siesser and Maness 2009; Schafer and Altevogt 2010; Cheng *et al*. 2011). A role of L1CAM in the regulation of proliferation may therefore be limited to non-neural cells. This conclusion is reinforced by the observation that in neuroblastomas, which are tumors of neural nature, expression of L1CAM was found associated not with severity, but with favorable outcomes (Wachoviak *et al*. 2007).

In all other investigated functions: adhesion to cell matrix proteins, migration, and neurite outgrowth, the role of L1CAM had been studied previously, however, in different cells and under different experimental conditions (Michelson *et al*. 2002; Buhusi *et al*. 2003; Cheng and Lemmon 2004; Schultheis *et al*. 2007; Dou *et al*. 2011; Kiefel *et al*. 2011). In our PC12 model, transfection of wtL1CAM was found to induce stimulatory effects in all cases, whereas transfection of the mutants induced smaller stimulatory or inhibitory effects depending on the function investigated. The mutation in the third Ig-like domain, E309K was in all cases the most inhibitory. The mutant of the forth FnIII-like repeat, P941L induced effects close to wt in terms of adhesion and migration, but exhibited significantly reduced NGF-induced neurite outgrowth. The latter function, in contrast, was clearly stimulated by the two mutants in the second Ig-like domain, H210Q and I219T. Therefore, the various mutants induce in neural cells different panels of defects. This conclusion, which may have a role in the well-known heterogeneity of the human L1 syndrome, agrees with previous findings of Schafer *et al*. (2010), based on the study of two ectodomain mutations distinct from ours, one in the second Ig-like domain, the other in the fifth FnIII-like repeat.

The second series of results concerned the coreceptor role of L1CAM in the transmembrane signaling of NGF. Functions of this type had been reported previously for other adhesion proteins of the Ig superfamily. In transfected Xenopus oocytes and in hippocampal neurons NCAM, the prototype of an Ig family distinct from that of L1CAM, was shown to bind and modulate the functioning of TrkB, establishing a complex with, and modulating the activity of inwardly rectifying K^+^ channels (Kleene *et al*. 2010). Another cell adhesion protein, ALCAM, has recently been shown to cooperate with NGF and thus to contribute to neuronal differentiation (Wade *et al*. 2012). As far as L1CAM, a coreceptor function increasing the ligand-induced tyrosine phosphorylation and neurite outgrowth had been reported, concerning, however, two growth factor receptors other than TrkA, epidermal growth factor (EGF) and fibroblast growth factor receptors (Islam *et al*. 2004; Kulahin *et al*. 2008). Interestingly, the coreceptor effect of L1CAM on the EGF receptor was dissipated by the E309K mutation (Nagaraj *et al*. 2009), the same that we have now found to inhibit the NGF-induced neurite outgrowth in both PC12-27 and PC12-TrkA cells. Previous studies had also reported a lack of reinforcement by L1CAM of the axon growth induced by NGF in cultured sensory neurons (Liu *et al*. 2002). This effect, however, depended on the small GTPase RhoA. Therefore, a coreceptor function of L1CAM on TrkA could not be excluded in neural cells with lower RhoA tone.

The use of the PC12-TrkA clone, where the responses to NGF are amplified by the high surface density of the receptor, was critical for the success of our experiments. The results obtained in PC12-27 and PC12-TrkA cells transfected with the various forms of L1CAM, and in PC12-TrkA cells incubated with the recombinant L1CAM-Fc chimeric protein, treated or not with NGF signaling blockers, revealed the protein to induce a positive modulation of the TrkA receptor signaling as a consequence of an interaction at the external surface of the cell. Whether this interaction is because of a direct L1CAM/TrkA binding could not be established because the coimmunoprecipitation attempts we have carried out so far were unsuccessful. This result suggests the interactions of L1CAM with TrkA (or with other proteins associated to the receptor) to be of low affinity, as previously demonstrated with the EGF receptor, where a coreceptor function of L1CAM is well established (Islam *et al*. 2004).

In conclusion, the specific investigation carried out in the high REST PC12 clones expressing only very low levels of the endogenous L1CAM has revealed new properties of the adhesion protein and of its mutants concerning, in particular, their differential functional effects and the coreceptor role with the NGF receptor, TrkA. The experimental, high REST PC12 cell model we have characterized could be used for the investigation of additional properties of L1CAM, important also for the pathology of the L1 syndrome, including the characterization of the interaction of L1CAM with the TrkA receptor, the mechanism of its coreceptor effect, and the putative defects of the interaction and modulation of TrkA typical of various L1CAM mutants.

## Abbreviations used

Ab: antibody
CRASH: Corpus callosum hypoplasia, mental Retardation, Adducted thumbs, Spastic paraplegia, and Hydrocephalus
DAPI: 4′-6-diamidino-2-phenylindole
DR: detergent-resistant
DS: detergent-soluble
ER: endoplasmic reticulum
Fn: fibronectin
Ig: immunoglobulin
L1CAM-Fc: recombinant, water-soluble chimera including the ectodomain of L1CAM and the Fc domain of human IgGs
L1CAM: L1 cell adhesion molecule
MTT: 3-(4,5-dimethylthiazol-2-yl)-2,5-diphenyltetrazolium bromide
NGF: nerve growth factor
P75-I: the TAT-Pep5 inhibitor of P75^NTR^
PBS: phosphate-buffered saline
REST: RE1-silencing transcription (factor)
TfR: transferring receptor
Trk**-**I: the TrkA inhibitor Calbiochem 648450
wt: wild-type

## Supporting information

**Figure S1.** PC12-27 transiently transfected with the L1CAM, wt and mutants: distribution at the surface and within the cell.

**Figure S2.** Recovery of L1CAM forms and of markers in the detergent-resistant (DR) plasma membrane domains from the transfected PC12-27 cells.

**Figure S3.** Proliferation of stably transfected PC12-27 cells.

As a service to our authors and readers, this journal provides supporting information supplied by the authors. Such materials are peer-reviewed and may be re-organized for online delivery, but are not copy-edited or typeset. Technical support issues arising from supporting information (other than missing files) should be addressed to the authors.

## References

[b1] De Angelis E, Watkins A, Schafer M, Brummendorf T, Kenwrick S (2002). Disease-associated mutations of L1CAM interfere with ligand interactions and cell surface expression. Hum. Mol. Genet.

[b2] Arevalo E, Shanmugasundararaj S, Wilkemeyer MF, Dou X, Chen S, Charness ME, Miller KW (2008). An alcohol binding site on the neural cell adhesion molecule L1. Proc. Natl Acad. Sci. USA.

[b3] Ballas N, Mandel G (2005). The many faces of REST oversee epigenetic programming of neuronal genes. Curr. Opin. Neurobiol.

[b4] Borgonovo B, Cocucci E, Racchetti G, Podini P, Bachi A, Meldolesi J (2002). Regulated exocytosis: a novel, widely expressed system. Nat. Cell Biol.

[b5] Bruce AW, Krejci A, Ooi L, Deuchars J, Wood IC, Dolezal V, Buckley NJ (2006). The transcriptional repressor REST is a critical regulator of the neurosecretory phenotype. J. Neurochem.

[b6] Buhusi M, Midkiff RR, Gates AM, Richter M, Schachner M, Maness PF (2003). Close homolog of L1 is an enhancer of integrin-mediated cell migration. J. Biol. Chem.

[b7] Chang MC, Wisco D, Ewers H, Norden C, Winckler B (2006). Inhibition of sphingolipid synthesis affects kinetics but not fidelity of L1/NgCAM transport along direct but not transcytotic axonal pathways. Mol. Cell. Neurosci.

[b8] Cheng L, Lemmon V (2004). Pathological missense mutations of neural cell adhesion molecule L1 affect neurite outgrowth and branching on an L1 substrate. Mol. Cell. Neurosci.

[b9] Cheng L, Wu Q, Guryanova OA, Huang Z, Huang Q, Rich JN, Bao S (2011). Elevated invasive potential of glioblastoma stem cells. Biochem. Biophys. Res. Commun.

[b10] Cocucci E, Racchetti G, Podini P, Meldolesi J (2007). Enlargeosome traffic: exocytosis triggered by various signals is followed by endocytosis, membrane shedding or both. Traffic.

[b11] D'Alessandro R, Klajn A, Stucchi L, Podini P, Malosio ML, Meldolesi J (2008). Expression of the neurosecretory process in PC12 cells is governed by REST. J. Neurochem.

[b12] Dityatev A, Bukalo O, Schachner M (2008). Modulation of synaptic transmission and plasticity by cell adhesion and repulsion molecules. Neuron Glia Biol.

[b13] Dou X, Menkari CE, Shanmugasundararaj S, Miller KW, Charness ME (2011). Two alcohol binding residues interact across a domain interface of the L1 neural cell adhesion molecule and regulate cell adhesion. J. Biol. Chem.

[b14] Eibl JK, Chapelsky SA, Ross GM (2010). Multipotent neurotrophin antagonist targets brain-derived neurotrophic factor and nerve growth factor. J. Pharmacol. Exp. Ther.

[b15] Gast D, Riedle S, Kiefel H, Müerköster SS, Schafer H, Schafer MK, Altevogt P (2008). The RGD integrin binding site in human L1-CAM is important for nuclear signaling. Exp. Cell Res.

[b16] Greene LA, Tischler AS (1976). Establishment of a noradrenergic clonal line of rat adrenal pheochromocytoma cells which respond to nerve growth factor. Proc. Natl. Acad. Sci. U S A.

[b17] Grundschober C, Malosio ML, Astolfi L, Giordano T, Nef P, Meldolesi J (2002). Neurosecretion competence. A comprehensive gene expression program identified in PC12 cells. J. Biol. Chem.

[b18] Islam R, Kristiansen LV, Romani S, Garcia-Alonso L, Hortsch M (2004). Activation of the EGF receptor kinase by L1-mediated homophilic cell interactions. Mol. Biol. Cell.

[b19] Itoh K, Fujisaki K, Watanabe M (2011). Human L1CAM carrying the missense mutations of the fibronectin-like type III domains is localized in the endoplasmic reticulum and degraded by polyubiquitylation. J. Neurosci. Res.

[b20] Jouet M, Rosenthal A, Armstrong G, MacFarlane J, Stevenson R, Paterson J, Metzenberg A, Ionasescu V, Temple K, Kenwrick S (1994). X-linked spastic paraplegia (SPG1), MASA syndrome and X-linked hydrocephalus result from mutations in the L1 gene. Nat. Genet.

[b21] Kamiguchi H (2003). The mechanism of axon growth: what we have learned from the cell adhesion molecule L1. Mol. Neurobiol.

[b22] Kenwrick S, Watkins A, De Angelis E (2000). Neural cell recognition molecule L1: relating biological complexity to human disease mutations. Hum. Mol. Genet.

[b23] Kiefel H, Pfeifer M, Bondong S, Hazin J, Altevogt P (2011). Linking L1CAM-mediated signaling to NF-kB activation. Trends Mol. Med.

[b24] Kleene R, Cassens C, Bähring R, Theis T, Xiao MF, Dityatev A, Schafer-Nielsen C, Döring F, Wischmeyer E, Schachner M (2010). Functional consequences of the interactions among the neural cell adhesion molecule NCAM, the receptor tyrosine kinase TrkB, and the inwardly rectifying K^+^ channel KIR3.3. J. Biol. Chem.

[b25] Kulahin N, Hinsby A, Kiselyov V, Berezin V, Bock E (2008). Fibronectin type III (FN3) modules of the neuronal cell adhesion molecule L1 interact directly with the fibroblast growth factor receptor. Mol. Cell. Neurosci.

[b26] Lazaridis I, Charalampopoulos I, Alexaki VI, Avlonitis N, Pediaditakis I, Efstathopoulos P, Calogeropoulou T, Castanas E, Gravanis A (2011). Neurosteroid dehydroepiandrosterone interacts with nerve growth factor (NGF) receptors, preventing neuronal fibronactinapoptosis. PLoS Biol.

[b27] Leoni C, Menegon A, Benfenati F, Toniolo D, Pennuto M, Valtorta F (1999). Neurite extension occurs in the absence of regulated exocytosis in PC12 subclones. Mol. Biol. Cell.

[b28] Lingwood D, Simons K (2010). Lipid rafts as a membrane-organizing principle. Science.

[b29] Liu RY, Schmid RS, Snider WD, Maness PF (2002). NGF enhances sensory axon growth induced by laminin but not by the L1 cell adhesion molecule. Mol. Cell. Neurosci.

[b30] Liu H, Focia PJ, He X (2011). Homophilic adhesion mechanism of neurofascin, a member of the L1 family of neural cell adhesion molecules. J. Biol. Chem.

[b31] Maness PF, Schachner M (2007). Neural recognition molecules of the immunoglobulin superfamily: signaling transducers of axon guidance and neuronal migration. Nat. Neurosci.

[b32] Marx M, Diestel S, Bozon M (2012). Pathomechanistic characterization of two exonic L1CAM variants located in trans in an obligate carrier of X-linked hydrocephalus. Neurogenetics.

[b33] Michelson P, Hartwig C, Schachner M, Gal A, Veske A, Finckh U (2002). Missense mutations in the extracellular domain of the human neural cell adhesion molecule L1 reduce neurite outgrowth of murine cerebellar neurons. Hum. Mutat.

[b34] Mikulak J, Negrini S, Klajn A, D'Alessandro R, Mavilio D, Meldolesi J (2012). Dual REST dependence of L1CAM: from gene expression to alternative splicing governed by Nova2 in neural cells. J. Neurochem.

[b35] Nagaraj K, Kristiansen lV, Skrzynski A, Castiella C, Garcia-Alonso L, Hortsch M (2009). Pathogenic human L1CAM mutations reduce the adhesion-dependent activation of EGFR. Hum. Mol. Genet.

[b36] Nakamura Y, Lee S, Haddox CL, Weaver EJ, Lemmon VP (2010). Role of the cytoplasmic domain of the L1 cell adhesion molecule in brain development. J. Comp. Neurol.

[b37] Needham LK, Thelen K, Maness PF (2001). Cytoplasmic domain mutations of the L1 cell adhesion molecule reduce L1-ankyrin interactions. J. Neurosci.

[b38] Panicker AK, Buhusi M, Thelen K, Maness PF (2003). Cellular signaling mechanisms of neural cell adhesion molecules. Front. Biosci.

[b39] Racchetti G, Lorusso A, Schulte C, Gavello D, Carabelli V, D'Alessandro R, Meldolesi J (2010). Rapid neurite outgrowth in neurosecretory cells and neurons is sustained by the exocytosis of a cytoplasmic organelle, the enlargeosome. J. Cell Sci.

[b40] Ratjen FG, Schachner M (1984). Immunocytological and biochemical characterization of a new neuronal cell surface component (L1 antigen) which is involved in cell adhesion. EMBO J.

[b41] Rawnaq T, Quaas A, Zander H (2012). L1 is highly expressed in tumors of the nervous system: a study of over 8000 human tissues. J. Surg. Res.

[b42] Roonprapunt C, Huang W, Grill R, Friedlander D, Grumet M, Chen S, Schachner M, Young W (2003). Soluble cell adhesion molecule L1-Fc promotes locomotor recovery in rats after spinal cord injury. J. Neurotrauma.

[b43] Rünker AE, Bartsch U, Nave KA, Schachner M (2003). The C264Y missense mutation in the extracellular domain of L1 impairs protein trafficking in vitro and in vivo. J. Neurosci.

[b44] Salton SRJ, Richter-Landsberg C, Greene LA, Schelanski ML (1983). Nerve growth factor-inducible large external glycoprotein: studies of a central and peripheral neuronal marker. J. Neurosci.

[b45] Schafer MK, Altevogt P (2010). L1CAM malfunction in the nervous system and human carcinomas Cell. Mol. Life Sci.

[b46] Schafer MK, Nam YC, Moumen A, Keglowich L, Bouché E, Küffner M, Bock HH, Rathjen FG, Raoul C, Frotscher M (2010). L1 syndrome mutations impair neuronal L1 function at different levels by divergent mechanisms. Neurobiol. Dis.

[b47] Schmid RS, Maness PF (2008). L1 and NCAM adhesion molecules as signaling coreceptors in neuronal migration and process outgrowth. Curr. Opin. Neurobiol.

[b48] Schulte C, Racchetti G, D'Alessandro R, Meldolesi J (2010). A new form of neurite outgrowth sustained by the exocytosis of enlargeosomes expressed under the control of REST. Traffic.

[b49] Schultheis M, Diestel S, Schmitz B (2007). The role of cytoplasmic serine residues on the adhesion molecule L1 in neurite outgrowth, endocytosis and cell migration. Cell. Mol. Neurobiol.

[b50] Siesser PF, Maness PF (2009). L1 cell adhesion molecules as regulators of tumor cell invasiveness. Cell Adh. Migr.

[b51] Suzuki T, Izumoto S, Fujimoto Y, Maruno M, Ito Y, Yoshimine T (2005). Clinico-pathological study of cellular proliferation and invasion in gliomatosis cerebri: important role of neural cell adhesion molecule L1 in tumour invasion. J. Clin. Pathol.

[b52] Tang N, Farah B, He M, Fox S, Malouf A, Littner Y, Bearer CF (2011). Ethanol causes the redistribution of L1 cell adhesion molecule in lipid rafts. J. Neurochem.

[b53] Tomasoni R, Negrini S, Fiordaliso S, Klajn A, Tkatch T, Mondino A, Meldolesi J, D'Alessandro R (2011). REST, TSC2 and β-catenin, interconnected in a signaling loop, govern proliferation and functions in PC12 neural cells. J. Cell Sci.

[b54] Vits L, Van Camp G, Coucke P (1994). MASA syndrome is due to mutations in the neural cell adhesion gene L1CAM. Nat. Genet.

[b55] Vos YJ, Hofstra RM (2010). An updated and upgraded L1CAM mutation database. Hum. Mutat.

[b56] Wachoviak R, Fiegel HC, Kaifi JT (2007). L1 is associated with favorable outcome in neuroblastomas in contrast to adult tumors. Ann. Surg. Oncol.

[b57] Wade A, Thomas C, Kalmar B, Terenzio M, Garin J, Greensmith L, Schiavo GP (2012). Activated leucocyte cell adhesion molecule modulates neurotrophin signaling. J. Neurochem.

[b58] Weller S, Gartner J (2001). Genetic and clinical aspects of X-linked hydrocephalus (L1 disease): mutations in the L1CAM gene. Hum. Mut.

[b59] Yamashita T, Tohyama M (2003). The p75 receptor acts as a displacement factor that releases Rho from Rho-GDI. Nat. Neurosci.

