# Supplementary figures and images for: L1CAM and its cell-surface mutants: new mechanisms and effects relevant to the physiology and pathology of neural cells
Source: J Neurochem. 2012 Dec 10;124(3):397–409. doi: 10.1111/jnc.12015 (PMC3557714; doi:10.1111/jnc.12015)

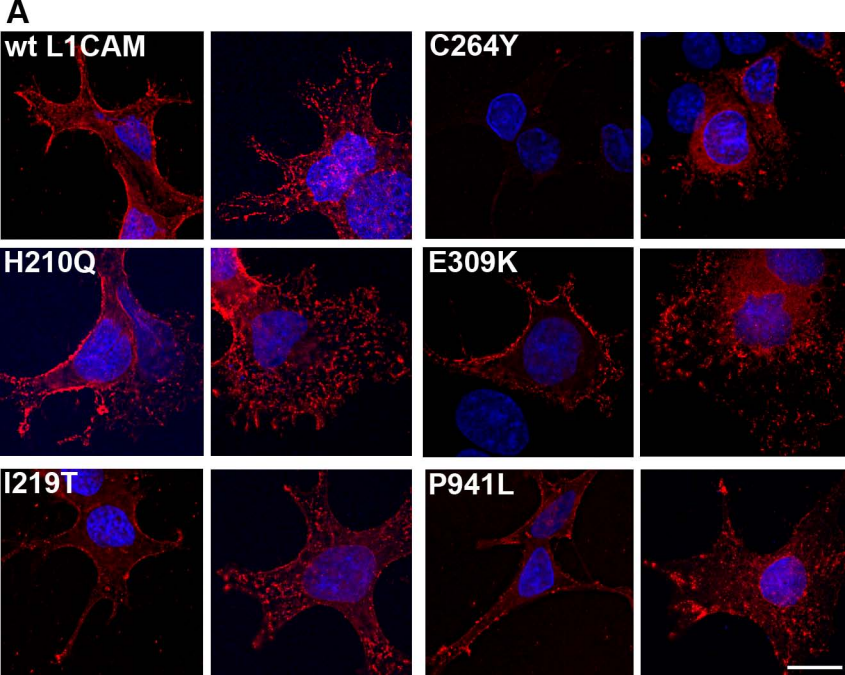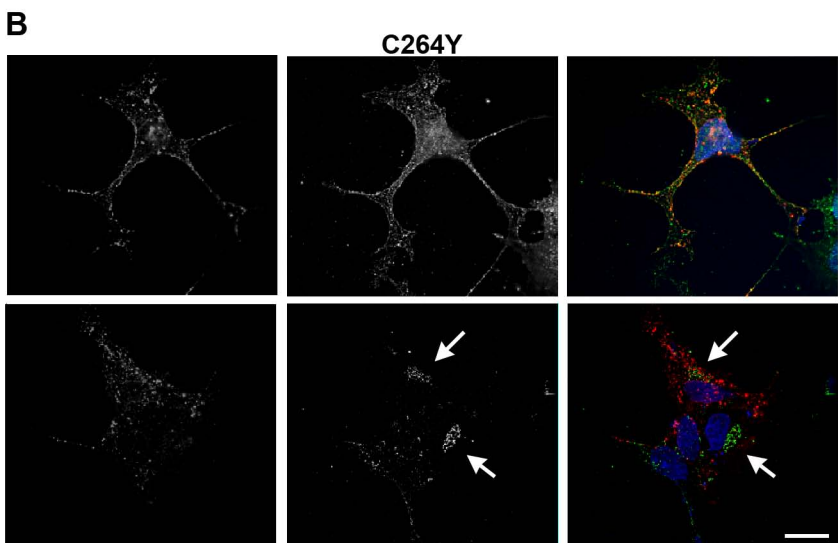

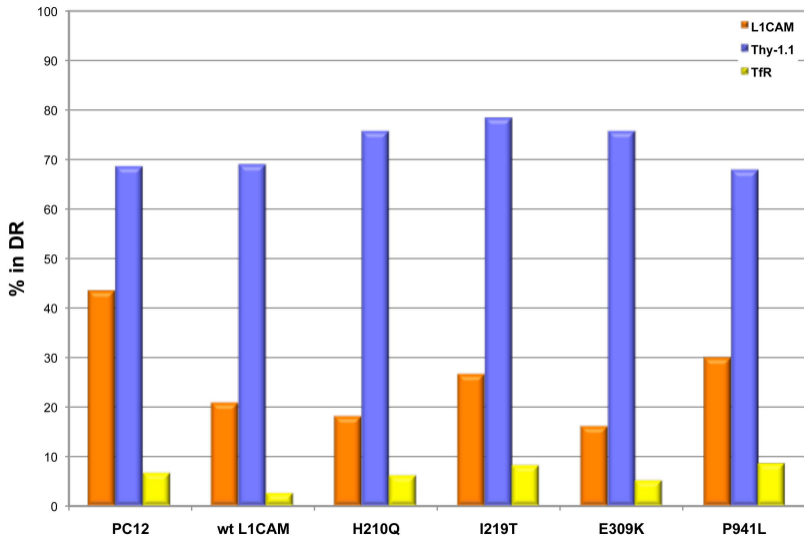

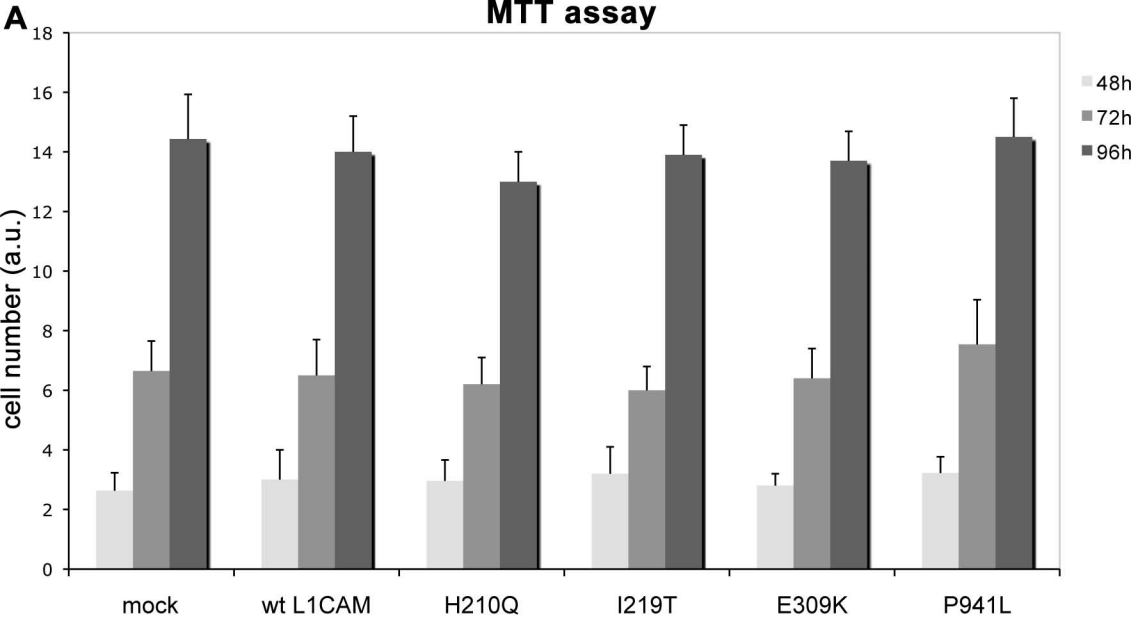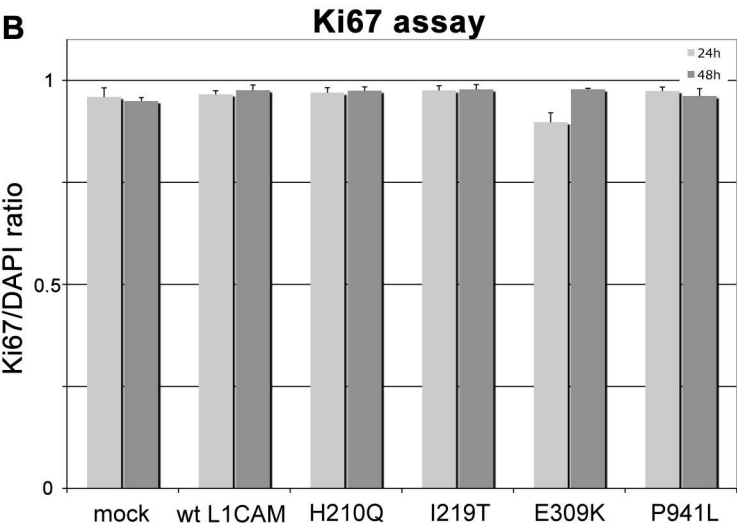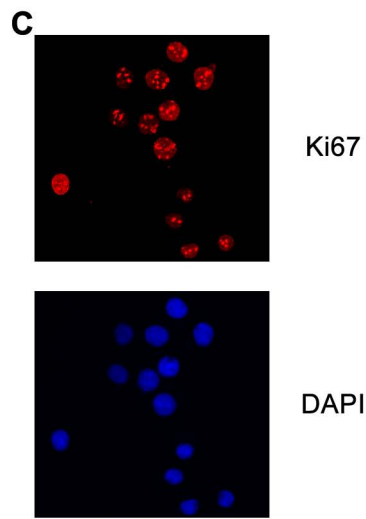

Supplement: Supplementary file 1 [file jnc0124-0397-SD1.pdf]

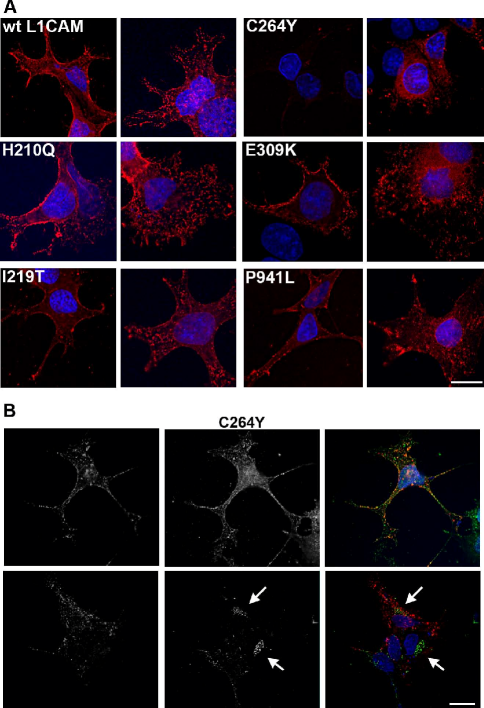

Supplement: Supplementary file 2 [file jnc0124-0397-SD2.png]
